# Using Technology to Improve Women's Health Care

**DOI:** 10.31486/toj.19.0113

**Published:** 2020

**Authors:** Logan Corey, Alex Vezina, Rajiv B. Gala

**Affiliations:** ^1^ Department of Obstetrics and Gynecology, Ochsner Clinic Foundation, New Orleans, LA; ^2^ The University of Queensland Faculty of Medicine, Ochsner Clinical School, New Orleans, LA

**Keywords:** *Education–medical*, *electronic health records*, *mobile applications*, *remote consultation*, *telemedicine*

## Abstract

**Background:** Technology is being integrated into all aspects of health care. While many applications offer novel experiences, the evidence supporting translation to improved education or care is evolving.

**Methods:** We review ways that technology is affecting a variety of fields pertinent to women's health, including patient communication, physician education, and health care performance.

**Results:** In the Ochsner Health Department of Obstetrics and Gynecology, we have developed a platform known as Connected Maternity Online Monitoring—Connected MOM—to encourage remote monitoring during the prenatal course. We are also assessing improvements in quality and safety through a centralized fetal heart rate monitoring bunker known as TeleStork.

**Conclusion:** Through systematic integration of technology into the delivery of women's health care at Ochsner, we hope to demonstrate sustainable improvements in physician skills, patient access, and quality and safety.

## INTRODUCTION

The primary goal of medicine has always been to provide the highest quality patient care. The method of delivering care has evolved from physicians making house calls to seeing patients in brick and mortar clinics, and care delivery will continue to change as other methods are discovered. Technology is being invented, repurposed, and optimized in every field of medicine, including obstetrics and gynecology, and women's health care is directly influenced by these technologies. We review ways that technology is affecting a variety of fields pertinent to women's health, including patient communication, physician education, and health care performance.

## ELECTRONIC HEALTH RECORDS

Electronic health records (EHRs) are digitalized databases of patient notes, objective measurements (eg, height, weight, vital signs), laboratory tests, images, and other health-related information, all centrally stored and accessible from the internet. Implementation of EHRs has been widespread because most insurance providers, including Medicare and Medicaid, mandate the use of electronic records for providers to receive full compensation.^1^ A key benefit of the EHR is the ability to retrieve data about specific patient populations in an integrated manner. For example, data sets can be created and stratified by categories such as race, age, diagnosis, and surgery, allowing providers to identify patients with certain problems for audit and evaluation. This capability is especially useful in women's health. For instance, patients with abnormal Pap smear or mammogram findings can be flagged to alert the physician to ensure proper management is followed. However, along with the benefits of the EHR are drawbacks. In a 2013 list of hazards published by the ECRI, 4 of the top 10 hazards were related to the use of EHRs: alert fatigue; increased ordering time; inflexible ordering formats; and automated/self-populating templates, leading to inaccuracies in medical records. Furthermore, reporting medical errors related to EHR systems is not mandatory.^2^

EHRs have contributed to the consolidation of health care at large monolithic centers. Although consolidated data and EHRs can increase patient safety, flow of data, and speed of record sharing,^3^ implementing this technology carries a significant burden. To internally manage both EHR and practice management technology, a practice needs to have computer programmers on staff as well as staff responsible for keeping up with changing regulations and compliance.^4^ Because of the investment required, many small practices have sought partnerships with larger entities such as hospitals or group practices. The changing landscape is also influenced by thin profit margins and the need for increased efficiency. EHRs provide increased efficiency in daily data collection, data entry, data processing, and the processing of claims and reimbursement, allowing for increased profits.^5^

Accessibility to health care includes receiving results of clinic and laboratory tests in a timely way, as well as prompt replies to patient inquiries. A major benefit of the EHR is the ability to rapidly communicate patient information. Most EHRs have designated patient portals that allow patients to securely log in and view messages from their physicians and laboratory results in real time. The patient portal also allows direct patient-to-physician communication that can help in clarifying diagnoses and prognoses and in answering general health-related questions. However, another EHR-related concern identified by the ECRI is possible Health Insurance Portability and Accountability Act (HIPAA) violations related to online and mobile phone access to patient data. Failure to maintain data in a secure, password-protected manner can result in privacy infractions and law violations, a major concern for many patients and providers.

## MOBILE HEALTH

Mobile health is the use of mobile devices, such as mobile phones, patient monitoring devices, and personal digital assistants, for medical and public health practice.^6^ Mobile health applications are varied and include software for fitness, weight, nutrition, smoking cessation, diabetic care, and medication use. Women's health is an especially attractive marketplace for creators of mobile health applications; more applications are available to support pregnancy than any other medical domain.

Applications generally take 7 to 12 months to launch publicly and cost an average of $270,000. As of 2015, more than 165,000 health-related applications were available in the Apple iTunes and Android application stores, and 34% of mobile phone owners had at least one health application on their mobile devices.^7^ The ability to create and make money from mobile applications far outpaces the ability of the scientific community to determine their efficacy and their effect on the improvement of measurable outcomes.

The mobile applications directly related to women's health are broad in topic and range from contraception to fertility to pregnancy guides. In August 2018, the US Food and Drug Administration (FDA) permitted marketing of the first mobile medical application for use in preventing pregnancy. The application contains an algorithm that calculates the days of the month a woman is likely to be fertile based on daily body temperature readings and menstrual cycle information, a method of contraception called fertility awareness.^8^ On the other end of the reproductive spectrum, Lopez et al introduced online-based lessons for postpartum women of varying socioeconomic levels on the benefits of breast feeding. Participants received a weekly text message and e-lessons for 6 months promoting breastfeeding, and questionnaires were used to quantify their progress. Fifty-seven percent of participants reported breastfeeding subjectively more from receiving daily text messages, and the rate of mothers exclusively breastfeeding increased from 48% to 57%.^9^

## REMOTE MONITORING AND TELEMEDICINE

Remote monitoring has the potential to remodel prenatal care by enabling more frequent contact with less inconvenience. Marko et al reported a 43% reduction in clinic visits, enhanced satisfaction, increased engagement, and no change in perinatal outcomes with remote monitoring. In their study, 100 first-trimester, low-risk patients were divided into 2 groups of 50. One group was assigned remote monitoring and decreased clinic visits (8 vs 14), and the other group received standard care. The remote monitoring group generated 5-fold more data points for weight and blood pressure measurements per patient.^10^ Ochsner Health has developed a similar system known as Connected Maternity Online Monitoring—Connected MOM. Expectant mothers who enroll in the program receive a tote bag with a wireless scale, a wireless blood pressure cuff, and an at-home urine protein kit. Patient data points are recorded at home and wirelessly uploaded to a HIPPA-secure system. Physicians can see the data points, and patients can contact physicians directly through the system, permitting communication between physicians and patients without clinic visits or phone calls. Variations of this model have reinforced the idea that mobile monitoring provides better care and more patient satisfaction than the standard of care,^10-12^ but objective data examining the effect of mobile monitoring on significant outcomes (eg, preterm birth, hypertensive disorders, intrauterine growth restriction) are needed. Patients can be easily taught how to use mobile monitoring systems, and most patients reported comfort with home monitoring skills, including measuring weight, blood pressure, and fetal heart tones.^13^

Beyond home monitoring, telemedicine has been integrated into the care of the pregnant patient. As early as 1997, Nores et al showed that the interpretation of first-trimester obstetric ultrasonography using a system of videotape review was equivalent to live video telemedicine.^14^ This practice has evolved and is commonly used at Ochsner Health, where many of the maternal fetal medicine ultrasounds are recorded by skilled technicians and evaluated later by the physician, allowing for increased efficiency without a decrease in quality.

Electronic fetal monitoring—surveillance of a fetus's heart rate with the goal of decreasing cerebral palsy, neonatal seizures, or intrapartum fetal death—can also be done remotely. Ochsner Health has implemented a system called TeleStork through which live, beat-to-beat monitoring is centralized, and heart tracings from laboring patients across the system are monitored. If the TeleStork monitors, usually trained registered nurses, note an abnormal fetal heart tracing, the physicians in-house are notified to ensure timely intervention. Although electronic fetal monitoring is subjective and open to interpretation and therefore difficult to standardize, it is still the most commonly used method of intrapartum surveillance during labor.^15^

## MEDICAL EDUCATION AND PERFORMANCE

Most current (2020) medical students and residents were born between 1977 and 1995 and belong to generation Y, the so-called Millennials. Their style of learning and personality traits are different from those of prior generations.^16^ Their learning preferences include simulation, interactive group activities, workshops, and game-style presentations of knowledge. Video capture systems used in surgical training of obstetrics and gynecology residents have been demonstrated to improve skillsets.^17^ High-fidelity simulations can be created to compensate for deficiencies in training. For instance, a contraception simulation developed for obstetrics and gynecology residents in Catholic hospitals included intrauterine device placement, Nexplanon implant, and Essure device placement and resulted in a 30% increase in knowledge.^18^ Auguste et al showed that for programs without video capture systems or large simulation centers, alternative technologies can provide equivalent teaching. The use of serious computer games is a viable education alternative, especially for electronic fetal monitoring interpretation.^19^

The use of mobile applications is just as prevalent in the obstetrics and gynecology provider population as in the patient population. A survey of obstetrics and gynecology residents in California showed that every respondent owned a mobile device (100% owned smartphones, and 74% owned tablets), and 95% of respondents used their mobile devices in clinical settings.^20^ Commonly used applications were pregnancy wheels, cervical cancer screening guidelines, and contraceptive eligibility guidelines. The American College of Obstetricians and Gynecologists application was the most common application used. Residents reported that they chose which applications to use based primarily on peer recommendation. Overall, 92% of respondents viewed mobile applications as a useful clinical tool, and 89% felt they improved efficiency.^20^ However, Patel et al reported a generational difference regarding comfort with mobile devices.^21^ They demonstrated that faculty were more likely to find smartphone-related behaviors in the clinical setting unprofessional compared to medical students.

New technology has also led to the development of innovative surgical devices. With beginnings in the National Aeronautics and Space Administration and Stanford University, the da Vinci Surgical System (Intuitive Surgical, Inc)—the first robotic surgical platform commercially available in the United States—was approved by the FDA in 2000 and has been used in gynecologic surgery since 2005.^22^ Da Vinci robotic surgery is widely implemented throughout Ochsner Health. As minimally invasive surgery becomes the standard for treatment of conditions as wide ranging as sacrocolpopexy and endometrial cancer, robotic surgery is increasingly being used. Length of stay, blood loss, complication rates, and objective cure rates are often comparable to traditional laparoscopic surgery with the benefit of ergonomic comfort for the surgeon.^23^ Despite these benefits, robotic surgery is often not optimally implemented. Desai et al identify administrative obstacles that have prevented widespread acceptance, including the steep learning curve, operational costs, and the culture of safety that surrounds the introduction of most new and disruptive technologies.^24^

## CONCLUSION

The progression of technology holds the promise of significant impact in the realm of women's health. Mobile technology and telemedicine can potentially allow for fewer in-person clinic visits for healthy patients, enhancing the accessibility of health care. Simulations and video-assisted feedback can help trainees develop skill in rare diseases and situations. Improvements in surgical technology can make procedures once thought to be operable only via open surgery or laparotomy routinely performed via minimally invasive routes. However, robust and reproducible data are needed as new technologies are introduced in women's health.

## Notes

This article meets the Accreditation Council for Graduate Medical Education and the American Board of Medical Specialties Maintenance of Certification competencies for Patient Care and Medical Knowledge.
